# Spatially Explicit Burden Estimates of Malaria in Tanzania: Bayesian Geostatistical Modeling of the Malaria Indicator Survey Data

**DOI:** 10.1371/journal.pone.0023966

**Published:** 2012-05-23

**Authors:** Laura Gosoniu, Amina Msengwa, Christian Lengeler, Penelope Vounatsou

**Affiliations:** 1 Department of Public Health and Epidemiology, Swiss Tropical and Public Health Institute, University of Basel, Switzerland; 2 Department of statistics, University of Dar es Salaam, Dar es Salaam, Tanzania; Université Pierre et Marie Curie, France

## Abstract

A national HIV/AIDS and malaria parasitological survey was carried out in Tanzania in 2007–2008. In this study the parasitological data were analyzed: i) to identify climatic/environmental, socio-economic and interventions factors associated with child malaria risk and ii) to produce a contemporary, high spatial resolution parasitaemia risk map of the country. Bayesian geostatistical models were fitted to assess the association between parasitaemia risk and its determinants. Bayesian kriging was employed to predict malaria risk at unsampled locations across Tanzania and to obtain the uncertainty associated with the predictions. Markov chain Monte Carlo (MCMC) simulation methods were employed for model fit and prediction. Parasitaemia risk estimates were linked to population data and the number of infected children at province level was calculated. Model validation indicated a high predictive ability of the geostatistical model, with 60.00% of the test locations within the 95% credible interval. The results indicate that older children are significantly more likely to test positive for malaria compared with younger children and living in urban areas and better-off households reduces the risk of infection. However, none of the environmental and climatic proxies or the intervention measures were significantly associated with the risk of parasitaemia. Low levels of malaria prevalence were estimated for Zanzibar island. The population-adjusted prevalence ranges from 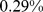 in Kaskazini province (Zanzibar island) to 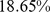 in Mtwara region. The pattern of predicted malaria risk is similar with the previous maps based on historical data, although the estimates are lower. The predicted maps could be used by decision-makers to allocate resources and target interventions in the regions with highest burden of malaria in order to reduce the disease transmission in the country.

## Introduction

Malaria is still a major public health problem in the United Republic of Tanzania, as the leading cause of inpatient and outpatient consultations. Ninety three percent of the population of Tanzania live in areas where malaria is transmitted for at least one month per year. Although Tanzania has been on the forefront in promoting the use of insecticide treated nets (ITNs), there are still between 60000 and 80000 malaria attributable deaths estimated per year, mainly children under the age of five ([1]). The disease is one of the main obstacles to the economical development of the country. The malaria situation in Zanzibar, the group of islands off the north-eastern coast of the Tanzania mainland, is a bit different than the one on the mainland. Over the past decade Zanzibar has reached very low levels of malaria endemicity due to rapidly scaling up of current antimalarial interventions([2]), and it is now one of the regions that is planning to eliminate the disease.

Planning and evaluating cost-effective strategies for the control and even more, the elimination of malaria, requires contemporary, high spatial resolution maps of the disease distribution as well as reliable estimates of the number of infected people. These measures will help tracking the progress and documenting reduction in parasitaemia rates as a result of control. Some earlier attempts to describe the situation of malaria transmission in Tanzania were based on analysis of historical parasite prevalence data ([3],[4]). However, historical field survey data were collected in different seasons and at non-standardized and overlapping age groups of the population. Therefore, accounting for seasonality and adjusting for age become challenging when modeling malaria survey data. The drawbacks of historical malaria survey data have been discussed in more details and addressed previously ([5], [6]). In addition, the estimated parasitaemia risk based on the analysis of historical data may not reflect the current malaria situation since they don't take into account the effect of malaria interventions which increased over the last decade. In addition, a map of endemic malaria distribution in Tanzania (available on MARA ([7]) website) was produced by [8] using spatially interpolated weather station data to define climatic suitability for malaria transmission.

From October 2007 to February 2008, the National Bureau of Statistics (NBS) in collaboration with the Office of the Chief Government Statistician (OCGS), Zanzibar implemented the Tanzania HIV/AIDS and Malaria Indicator Survey (THMIS). The aims of this survey were to assess the prevalence of HIV infection among Tanzanian adults and the prevalence of malaria infection and anemia among children under five years old. Here Bayesian geostatistical models based on environmental and climatic risk factors were implemented to produce the first contemporary, high spatial resolution parasitaemia risk map and spatially explicit disease burden estimates for Tanzania by analyzing the THMIS malaria data.

## Materials and Methods

### 1.2 THMIS Data

#### Ethic statement

The survey protocol was submitted to and approved by the National Institute for Medical Research (NIMR). Written informed consent was obtained from the parent or guardian of the child. The statement explained the purpose of the test, how the test would be administered, and advised the parent or guardian that the results would be available as soon as the test was completed. Finally, permission was requested for the test to be carried out.

**Figure 1:**
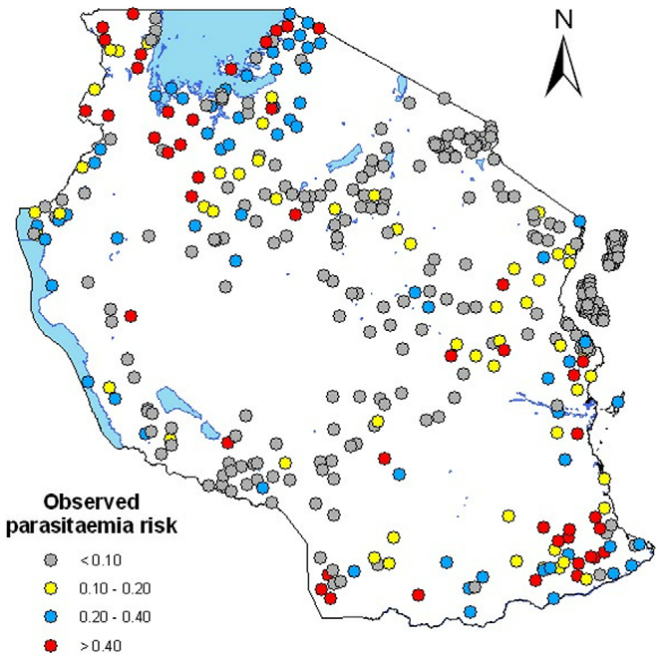
Observed parasitaemia prevalence in children less than 5 years old from the THMIS carried out 465 locations.

The THMIS was conducted over a four-month period, from October 2007 until February 2008. A two-stage sampling approach was used to select the surveyed population. In the first stage were selected 475 clusters consisting of enumeration areas defined for the 2002 Population and Housing Census. In particular, 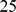 sample points were selected in Dar es Salaam, 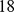 in each of the other 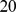 regions in the mainland and 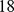 sample points in each of the 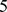 regions in Zanzibar. The second stage of selection involved the systematic sampling of households from the clusters. A total of 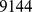 households throughout Tanzania were sampled, 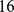 from each sampling point in Dar es Salaam, 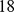 households per cluster in the other regions in mainland and Pemba and 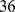 households per sampling unit in Unguja. Further details on the sampling approach can be found in [9]. In the selected households men and female age 15–49 were interviewed individually. Basic demographic information and data on the health status of the person interviewed and other members of the household were collected. In addition, information on the characteristics of the household dwelling, such as source of water, type of toilet facilities, materials used to construct the house and possession of various durable goods were gathered. Based on the household characteristics and household possessions a wealth index was constructed. The THMIS assembled also information on household coverage of malaria interventions such as IRS activities and ownership/use of bednets or ITNs. In addition, blood samples for anemia and malaria testing among children 6–59 months were collected. Although there is a large amount of information on HIV/AIDS knowledge and behavior and HIV prevalence, the analysis of these data is not the focus of this paper. The sampled clusters where parasitaemia prevalence data were collected are shown Figure 1.

### 1.3 Environmental and climatic data

Transmission of malaria is environmentally driven, therefore climatic and environmental proxies were used as parasitaemia predictors. The following climatic and environmental factors were used in the analysis: land surface temperature (LST), rainfall, normalized difference vegetation index (NDVI), altitude and distance to nearest permanent water body. The sources of these data, as well as the spatial and temporal resolution are shown in Table 1. Further details on the description of the climatic factors are given in [10]. The environmental factors with available temporal resolution (LST, rainfall and NDVI) were acquired for the one year period previous to the survey and annual average of each environmental covariate was calculated and extracted at each data location.

**Table 1:** Spatial databases used in the analysis.

Factor	Spatial	Temporal	Source
	Resolution	Resolution	
LST	1 km 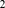	8 days	MODIS
			http://lpdaac.usgs.gov/modis/myd11c1v4.asp
Rainfall	8 km 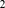	10 days	ADDS
			http://earlywarning.usgs.gov/adds/
NDVI	1 km 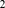	16 days	MODIS
			http://lpdaac.usgs.gov/modis/mod13q1v4.asp
Altitude	1 km 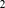	N.A.	USGS-DEM
Permanent water bodies	1 km 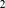	N.A.	Health Mapper
			http://eros.usgs.gov/products/elevation/dem.html

### 1.4 Statistical modeling

At each location in 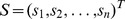 let us consider the binary outcome 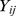 which takes value 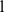 or 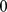 to indicate whether the child 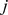 at location 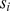 was found parasitaemia positive. A logistic regression model was used to relate the outcome to its predictors. The multivariate logistic regression model is given by
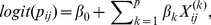
(1)where 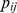 is the risk of child 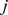 at location 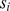 of having parasitaemia, 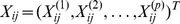 are the covariates and 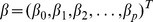 is the vector of regression coefficients, including the intercept. Preliminary frequentist statistical analysis was performed in Stata version 10.0 (Stata corporation, College Station, TX) to identify the covariates significantly associated with the parasitaemia prevalence and to check for possible nonlinear trends of the relationship between explanatory variables and response variable. To account for the nonlinear relationship between malaria and demographic or environmental and climatic covariates, the continuous variables were categorized, where the cutoffs points were chosen based on outcome-covariate scatter plots.

#### 1.4.1 Bayesian geostatistical modeling

The model described above does not consider the spatial relationship among the parasitaemia survey locations. The standard way of incorporating the geographical dependence in the model is by introducing spatially correlated random effects 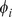 at every sampled location 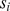. Using the geostatistical design described in [11] the underlying spatial process was modeled by the residuals via a multivariate Normal distribution with mean 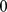 and the covariance matrix 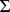, 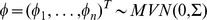. The covariance matrix is defined as a function which represents the decay in correlation between pairs of locations with distance. For this analysis, an exponential correlation function was chosen, that is 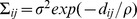, where 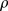 describes how fast the spatial correlation declines with distance between locations 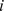 and 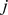 and 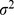 represents the variance of the spatial process. Measurement error or microscale variations are modeled by the independent and normally distributed random effects 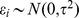, where 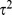 is interpreted as a nugget effect. The model in (1) can be written
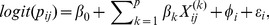
(2)This type of hierarchical models are usually fitted within a Bayesian framework because it allows flexible modeling and inference and avoids the computational problems met in likelihood-based fitting. The trade-off for the flexibility of a fully Bayesian approach is the complexity of the model fit. This step is carried out via the implementation of Markov chain Monte Carlo methods. To complete the specification of the Bayesian hierarchical model, prior distributions need to be assigned to the model parameters 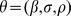. Further, Bayesian inference is based on the posterior distribution, that is the conditional distribution over parameters given observed data. Non-informative Normal prior distributions were specified for the intercept and the regression coefficients, 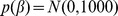. The spatial correlation parameters 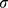 and 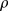 were assigned an inverse gamma and a gamma prior distribution, respectively, 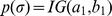 and 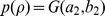. Non-informative inverse gamma prior distribution was chosen for the non-spatial variance, 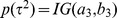. The values of the parameters 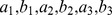 were chosen such that the mean of the corresponding distribution is 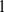 and the variance 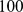. A two chain sampler of 100000 iterations was run with a burn-in of 10000 iterations and the convergence was assessed by examining the ergodic averages of selected parameters.

Separate geostatistical models were fitted for mainland Tanzania and the two islands covering Zanzibar (Pemba and Unguja). In particular, for each region we fitted Bayesian geostatistical models to assess the effects of interventions after adjusting for climatic and environmental factors and to estimate the disease burden at high spatial resolution. Model A includes as covariates the demographic variables, socio-economic status and malaria interventions, model B includes only the environmental and climatic proxies and model C includes the demographic variables, socio-economic status, malaria interventions and environmental/climatic factors. For each model, a burn-in of 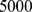 iterations was run. Convergence was assessed by inspection of the ergodic averages for all parameters and was achieved after 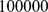 iterations for all models. Samples obtained from MCMC are not independent but autocorrelated, therefore a thinning of 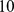 iterations was used when extracting samples from the posterior distribution. Disease burden estimates were obtained using Bayesian kriging and employing only environmental predictors (model B) because malaria intervention data and wealth index information are not available for the whole country. In particular, predictions were made at 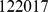 locations covering mainland Tanzania, 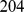 locations covering Unguja and 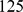 locations covering Pemba. The Bayesian kriging was implemented using software written by the authors in Fortran 95 (Compaq Visual Fortran Professional 6.6.0) using standard numerical libraries (NAG, The Numerical Algorithms Group Ltd.). The number of children infected with malaria parasites were obtained by combining the number of children with the parasitaemia risk at each of the 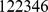 locations. The population data were acquired from the International Data Base of the U.S. Census Bureau, Population Division for the year 2006. Although these data are outdated and maybe underestimate the total population in the country, they represent the only source of population data at such small spatial resolution.

To validate model B, which was used to predict parasitaemia risk, model fit was carried out on a randomly selected subset of 299 locations (training set). The remaining 75 locations (test points), comprising a simple random sample, were used for validation. To assess the predictive performance of the model, 11 credible intervals of the posterior predictive distribution at the test locations with probability coverage equal to 5%, 10%, 20%, 30%, 40%, 50%, 60%,70%, 80%, 90% and 100%, respectively were calculated and the percentages of test locations with observed parasitaemia prevalence falling in these intervals were computed.

## Results

A total of 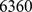 children under 5 years old at 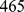 geo-located clusters had *P. falciparum* parasitaemia results from blood film examination. The prevalence of asymptotic malaria among children in Tanzania was estimated from the pooled data at 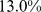 (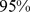 CI: 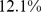
–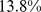). In mainland the parasitaemia risk at cluster level ranged from 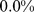 to 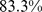 with a mean of 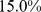. 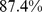 of clusters had malaria risk below 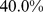. The malaria risk in Zanzibar island was much lower, varying from 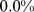 to 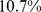 in Pemba and 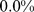 to 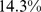 in Unguja. In terms of malaria intervention coverage, there is a big discrepancy between mainland and Zanzibar islands. In particular, 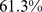 of households in mainland and 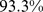 of households in Zanzibar had at least one bednet used for sleeping (p-value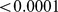). The difference is even bigger when it comes to IRS coverage. In mainland, only 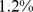 of household had walls sprayed with insecticide, while in Zanzibar island the proportion was 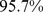 (p-value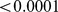).

The estimates of the effect of demographic, wealth index, malaria interventions and environmental/climatic factors on parasitaemia risk obtained from the bivariate and multivariate non-spatial models in mainland Tanzania are presented in Table 2. The bivariate analyses indicates that in mainland all predictors except malaria interventions were statistical significantly associated with the parasitaemia risk at 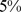 significance level. In particular, children living in urban areas had significantly lower risk of parasitaemia compared with children in rural areas. A positive relationship with age indicated that the older the child the higher the risk of malaria. As it was expected, the wealth index was negatively associated with the parasitaemia risk, suggesting that children belonging to better-off households had less risk of acquiring malaria. Both malaria intervention variables included in this analysis (bednet ownership and IRS) showed a non-protective effect, although none of them were statistically significant. Altitude was negatively associated with the parasitaemia risk, indicating that children at above 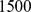 meters had lower odds of malaria. Surprisingly, the odds of malaria for children living further away from water bodies were higher than the odds of children closer to permanent sources of water. The results also suggest that the malaria odds are increasing with an increase in rainfall, vegetation index, day and night temperature. In Pemba, the relationship between parasitaemia and age and wealth index is not monotone, as in mainland. In addition, we observe a protective effect of bednets, which is not significant. Bivariate analysis suggested that in Unguja none of the covariates under study were significantly associated with parasitaemia prevalence.

**Table 2:** Association of parasitaemia risk with demographic variables, socio-economic status, malaria interventions and environmental/climatic factors in mainland Tanzania resulting from the bivariate and multivariate non-spatial models.

Variable	Bivariate		Multivariate	
	non-spatial model		non-spatial model	
	OR	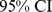	OR	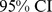
*Residence* (Rural)	1.0		1.0	
Urban	0.23	(0.17,0.33)	0.50	(0.34,0.74)
*Age* ( 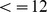 months)	1.0		1.0	
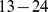	1.70	(1.25,2.33)	1.75	(1.27,2.42)
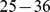	2.26	(1.67,3.07)	2.49	(1.81,3.42)
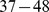	2.28	(1.68,3.11)	2.49	(1.80,3.43)
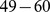	2.63	(1.94,3.56)	2.94	(2.14,4.05)
*Wealth index* (Most poor)	1.0		1.0	
Very poor	0.99	(0.81,1.22)	0.89	(0.71,1.10)
Poor	0.87	(0.71,1.07)	0.81	(0.65,1.02)
Less poor	0.52	(0.40,0.66)	0.55	(0.42,0.72)
Least poor	0.15	(0.10,0.24)	0.28	(0.17,0.45)
*Having bednet* (No)	1.0		1.0	
Yes	1.12	(0.96,1.32)	1.03	(0.86,1.23)
* IRS* (No)	1.0		1.0	
Yes	1.19	(0.61,2.32)	1.03	(0.50,2.15)
*Altitude* ( 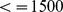 m)	1.0		1.0	
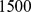	0.28	(0.21,0.37)	0.43	(0.30,0.61)
*Distance to nearest water body* ( 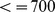 m)	1.0		1.0	
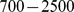	1.41	(1.09,1.83)	1.43	(1.09,1.89)
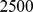	1.63	(1.27,2.09)	1.67	(1.28,2.18)
*Rainfall* ( 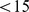 mm)	1.0		1.0	
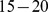	2.69	(1.92,3.76)	1.86	(1.30,2.65)
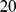	4.46	(3.24,6.14)	2.80	(1.99,3.94)
*NDVI* ( 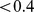 )	1.0		1.0	
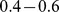	4.15	(3.25,5.29)	2.70	(2.07,3.51)
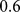	4.42	(3.23,6.05)	2.68	(1.83,3.94)
*Day temperature* ( 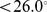 C)	1.0		1.0	
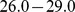	2.76	(2.01,3.80)	2.15	(1.53,3.03)
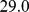	1.76	(1.30,2.39)	1.65	(1.15,2.38)
*Night temperature* ( 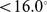 C)	1.0		1.0	
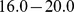	2.12	(1.74,2.59)	1.67	(1.27,2.20)
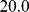	1.58	(1.21,2.07)	1.52	(1.08,2.12)

Table 3 presents the effect of residence, age, wealth index, malaria interventions and environmental/climatic factors on parasitaemia risk in mainland. These estimates were obtained by fitting Bayesian geostatistical models described in section 2.3.1. After taking into account the spatial correlation, residence (living in urban area has a protective effect), age (older children are more likely than younger children to test positive for malaria) and wealth index (living in better-off households has a protective effect) remained significantly associated with malaria. This was not the case for the environmental and climatic proxies. When all covariates were included in the model, only residence, age, wealth index (less poor, least poor) remained statistically significant associated with parasitaemia risk in mainland Tanzania (Table 3).

**Table 3:** Association of parasitaemia risk with demographic variables, socio-economic status, malaria interventions and environmental/climatic factors in mainland Tanzania resulting from the Bayesian geostatistical models.

Variable	Geostatistical		Geostatistical		Geostatistical	
	model A*		model B**		model C***	
	OR	95% BCI	OR	95% BCI	OR	95% BCI
*Residence* (Rural)	1.0				1.0	
Urban	0.36	(0.20,0.62)			0.42	(0.24,0.74)
*Age* ( 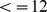 months)	1.0				1.0	
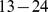	1.89	(1.34,2.68)			1.88	(1.33,2.69)
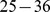	2.64	(1.88,3.74)			2.64	(1.87,3.74)
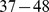	2.63	(1.87,3.72)			2.64	(1.87,3.75)
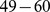	3.41	(2.43,4.83)			3.44	(2.43,4.88)
*Wealth index* (Most poor)	1.0				1.0	
Very poor	1.07	(0.83,1.37)			1.07	(0.83,1.37)
Poor	0.95	(0.73,1.24)			0.95	(0.73,1.24)
Less poor	0.69	(0.50,0.95)			0.70	(0.51,0.96)
Least poor	0.34	(0.20,0.59)			0.37	(0.20,0.63)
*Having bednet* (No)	1.0				1.0	
Yes	0.95	(0.77,1.16)			0.92	(0.75,1.13)
*IRS* (No)	1.0				1.0	
Yes	1.42	(0.42,4.28)			1.17	(0.33,3.63)
*Altitude* ( 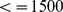 m)			1.0		1.0	
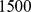			0.53	(0.25,1.04)	0.51	(0.25,1.02)
*Distance to nearest water body* ( 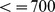 m)			1.0		1.0	
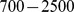			1.34	(0.82,2.24)	1.35	(0.82,2.23)
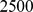			1.39	(0.84,2.50)	1.40	(0.86,2.28)
*Rainfall* ( 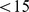 mm)			1.0		1.0	
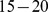			0.76	(0.36,1.60)	0.97	(0.48,1.90)
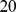			1.28	(0.47,2.99)	1.43	(0.63,3.14)
*NDVI* ( 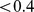 )			1.0		1.0	
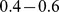			1.79	(1.13,2.91)	1.47	(0.88,2.45)
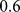			1.90	(0.89,3.89)	1.40	(0.67,2.98)
*Day temperature* ( 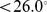 C)			1.0		1.0	
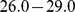			2.09	(1.14,3.87)	2.01	(1.13,3.60)
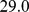			0.94	(0.49,1.82)	1.07	(0.56,2.03)
*Night temperature* ( 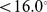 C)			1.0		1.0	
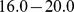			1.46	(0.83,2.63)	1.47	(0.81,2.73)
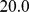			1.20	(0.58,2.42)	1.31	(0.61,2.81)

* The model includes only the demographic variables, socio-economic status and malaria interventions as predictors.

** The model includes only the environmental and climatic proxies as predictors.

*** The model includes the demographic variables, socio-economic status, malaria interventions and environmental/climatic factors as predictors.

Posterior estimates of the spatial parameters (spatial variance and decay parameter) and the non-spatial variance are presented in Table 4. In all three areas the spatial variance was significantly larger than the non-spatial variance. The estimates of the range parameter suggest a strong spatial correlation in mainland and a weak spatial correlation in Pemba and Unguja.

**Table 4:** Posterior estimates of spatial parameters.

Spatial parameter	Tanzania		Pemba		Unguja	
	Median	95% BCI^a^	Median	95% BCI^a^	Median	95% BCI^a^
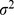	1.74	(0.63, 148.4)	3.23	(0.48, 40.5)	2.89	(0.41, 95.68)
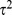	0.59	(0.22, 0.98)	1.47	(0.28, 21.65)	1.49	(0.29, 31.5)
range^b^	206.25	(124.53, 370.79)	1.05	(0.52, 21.22)	0.67	(0.34, 17.41)

a : Bayesian confidence intervals.

b : Based on the decay parameter 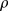, the range parameter 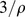 (in km) is calculated.

**Figure 2:**
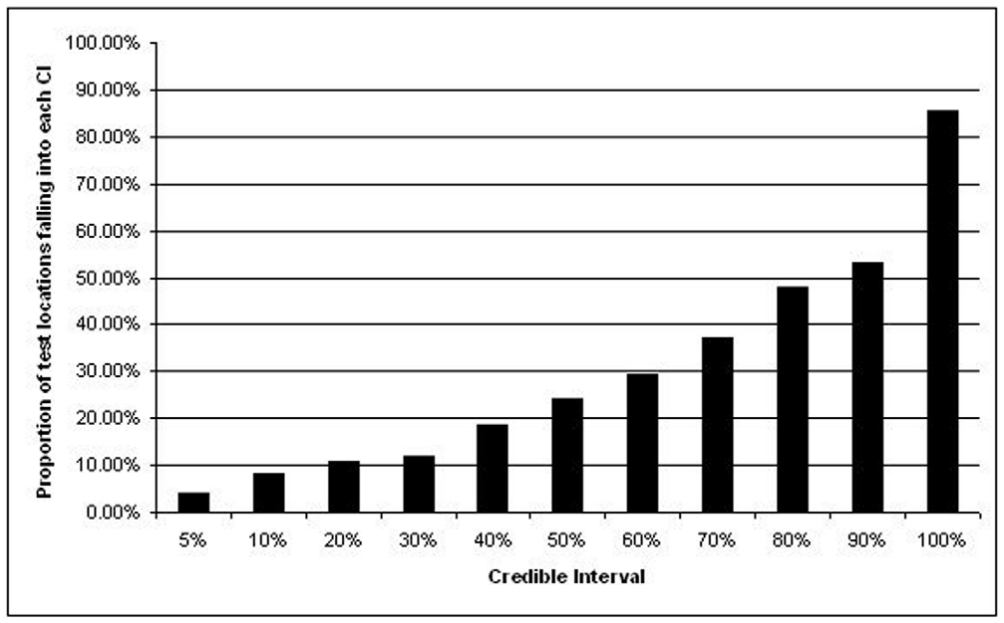
Percentage of test locations with observed malaria prevalence falling within the 5%, 10%, 20%, 30%, 40%, 50%, 60%, 70%, 80%, 90% and 100% credible intervals of the posterior predictive distribution.

Figure 2 shows the prediction performance of model B via proportion of test locations with malaria prevalence falling into credible intervals of coverage ranging from 5% to 100%. Based on these results we can state that the model is fairly accurate, with 4%, 53% and 85.33% of the test locations within the 5%, 90% and 100% credible interval respectivelly.

**Figure 3:**
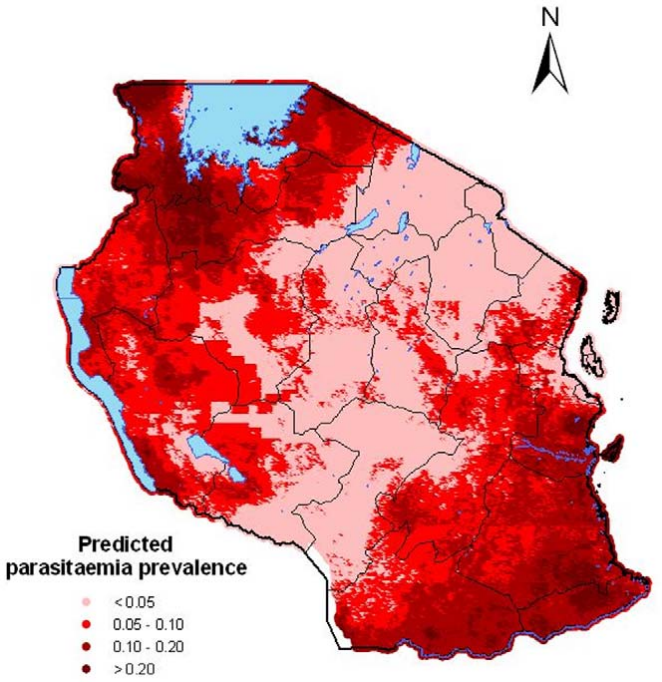
Smooth map of the parasitaemia risk in children 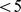 years in Tanzania.

**Figure 4:**
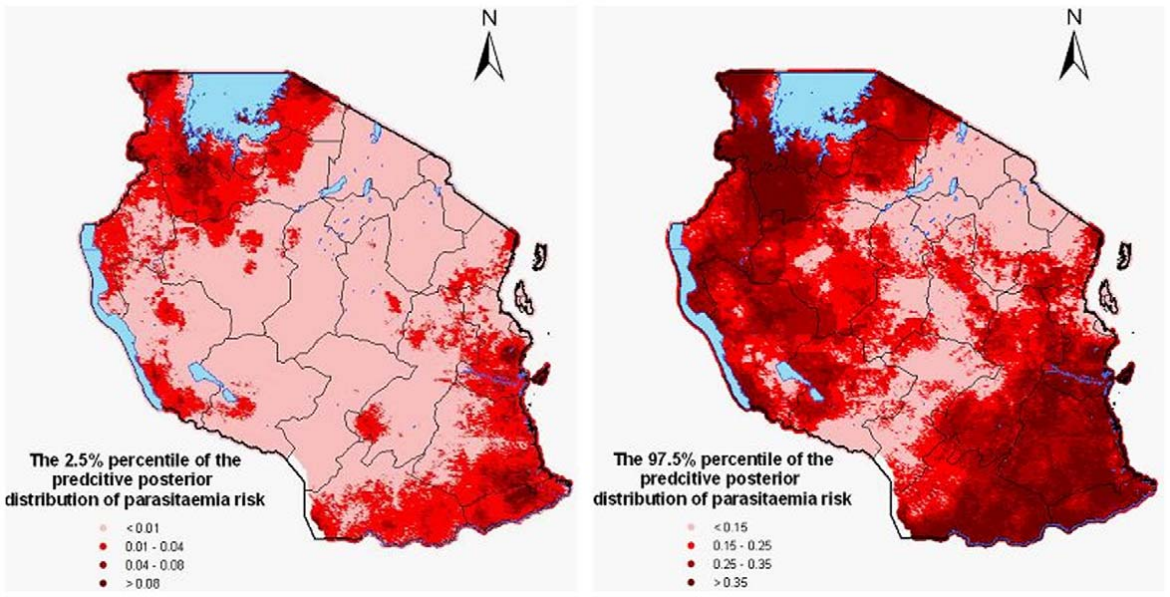
The 2.5% (left) and 97.5% (right) percentiles of the predicted posterior distribution for the parasitaemia prevalence.

The map of predicted parasitaemia risk for the United Republic of Tanzania is shown in Figure 3. The risk of malaria varies from 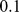
% to 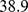
%, with a median of 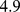
%. High levels of prevalence (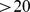
%) were predicted in the north of the country, the area around Lake Victroia (regions Kagera, Mara and Shinyanga) as well as in the south of the country (provinces Pwani, Lindi, Mtwara and Ruvuma). Low levels of parasitaemia risk (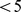
%) are observed in the central part of the country, north-east and south-west and the island of Zanzibar. The lower (2.5%) and upper (97.5%) percentiles of the posterior distribution corresponding to the predicted malaria risk are depicted in Figure 4.

**Figure 5:**
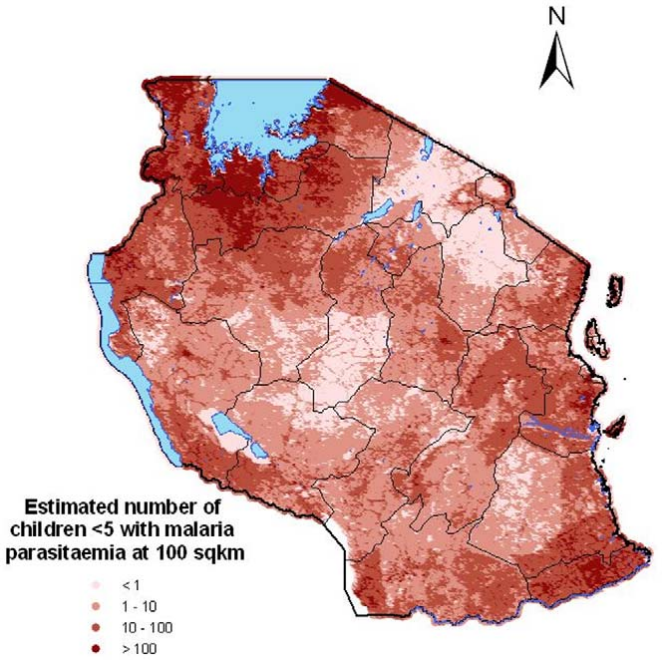
Estimated number of children 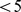 years infected with malaria parasite in Tanzania.

Figure 5 shows the predicted number of children with malaria parasites in the United Republic of Tanzania. The estimates of number of children 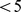 with malaria parasites at the regional level are presented in Table 5. We observe that both before and after adjusting for population distribution, Kaskazini region in Unguja had the lowest risk of malaria and Mtwara region in mainland had the highest risk.

**Table 5:** Estimates of the number of children less than 5 years old with malaria parasites at regional level.

Region	Number of	Infected	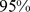 CI	Model-based	Model-based prevalence
	children 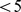	children		prevalence	adjusted for population
Arusha	243725	1694	(200,11362)	1.11%	0.70%
Dar-Es-Salaam	422267	6203	(2180,15486)	3.05%	1.47%
Dodoma	287515	6895	(849,37505)	2.70%	2.40%
Iringa	251918	5374	(610,30768)	2.14%	2.13%
Kagera	349344	58494	(13291,122501)	17.68%	16.74%
Kaskazini-Pemba	31049	366	(105,1152)	1.04%	1.18%
Kaskazini-Unguja	25086	73	(16,263)	0.32%	0.29%
Kigoma	286895	24568	(4423,79297)	7.78%	8.56%
Kilimanjaro	216248	1287	(162,8223)	0.60%	0.59%
Kusini Unguja	14436	163	(41,595)	0.99%	1.13%
Kusini-Pemba	29202	263	(81,864)	0.77%	0.90%
Lindi	130174	22810	(5931,48259)	12.82%	17.52%
Manyara	170711	2535	(328,14111)	1.37%	1.49%
Mara	232267	36158	(12153,19547)	11.26%	15.57%
Mbeya	349050	9053	(1186,45019)	3.47%	2.59%
Mjini-Magharibi	44467	386	(127,1035)	0.76%	0.87%
Morogoro	304225	17724	(2621,72404)	6.01%	5.83%
Mtwara	192776	35946	(10332,71287)	17.96%	18.65%
Mwanza	448578	55631	(13867,130405)	13.13%	12.40%
Pwani	155518	17365	(4362,43447)	11.10%	11.17%
Rukwa	192567	13998	(2529,48138)	6.62%	7.27%
Ruvuma	193856	18826	(3478,55652)	10.64%	9.71%
Shinyanga	492062	56444	(12798,144062)	10.97%	11.47%
Singida	191261	4438	(596,24707)	2.21%	2.32%
Tabora	288518	12991	(2090,53399)	4.82%	4.50%
Tanga	265337	9591	(1671,37409)	4.44%	3.61%
TOTAL	5809052	419277	(96028,1170757)	5.99%	7.22%

## Discussion

The parasitaemia survey of the 2007–08 THMIS was designed to provide an estimate of the malaria prevalence among children under five and to measure malaria prevention and treatment outcomes including possession and use of ITNs and IRS activities in the United Republic of Tanzania. Parasitaemia data of this survey were modeled within a spatial context to identify predictors significantly associated with malaria and to produce the 2008 malaria risk map as well as the map of infected children under five in the United Republic of Tanzania. The geostatistical model had a high predictive ability as shown by the high percentage of test locations with observed parasitaemia prevalence falling in the credible intervals. Previous efforts ([12], [3],[4]) have been made to map malaria prevalence in Tanzania, by collecting and analyzing historical survey data. The risk map presented here depicts with high-fidelity the contemporary situation of malaria in the country and could be used as part of monitoring and evaluation of malaria situation and ongoing interventions in the country.

Compared to the distribution maps based on historical data([3],[4]), our malaria risk map shows an overall lower prevalence over the country. The malaria distribution map shows very low levels of prevalence (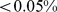) on the island of Zanzibar. This could be explained by the increase in coverage levels of interventions which significantly reduced the burden of disease [13]. This type of interventions should be emulated in mainland Tanzania, especially in the areas of high risk (provinces Kagera, Mara, Shinyanga, Pwani, Lindi, Mtwara and Ruvuma). Although we observe a decrease in malaria prevalence over the last years, the magnitude of the decline is not clear in the absence of a baseline map. There are, however, evidences from other studies such as the National Malaria Control Programme (NMCP) survey carried out in 21 districts which shows a decline of 30% in under-five malaria risk between 2006 and 2008. In Ifakara and Rufiji Demographic Surveillance System (DSS) all-age malaria prevalence has dropped 50%–60% between the years 2000 and 2008 (Impact and ALIVE projects, Ifakara Health Institute, unpublished). These reports are also supported by reduction in the intensity of malaria transmission by about 80% between 1990 and 2001–2003 ([14]) due mainly to increase of bednets and ITNs use. A comprehensive review of the impact of malaria control in Tanzania can be found in [15] who concluded that malaria is “down but not out”.

Intervention measures (bednet, IRS) seem to have no statistically significant effect on malaria risk. These results could be explained, on one hand, by the fact that only data on bednet ownership/use were collected, without distinguishing between treated and untreated nets. Since untreated nets are less effective than treated ones ([16]), this had certainly influence the results of the risk factor analysis. On the other hand, usage of mosquito nets, especially by children is very important, but not all owned bednets were also used. The other important factor is that the IRS coverage was very low for the mainland (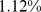), therefore it is difficult to observe a significant effect, especially if the implementation of the intervention is not fully correct. The absence of significant association between intervention measures and malaria risk may be explained by other factors such as indication bias (children living in high risk areas use more frequently the intervenition measures), exophilic behavior of vectors, resistance of vectors to the insecticides and lack of efficacy of these interventions.

In the multivariate non-spatial model for mainland Tanzania demographic (residence and age), wealth index and environmental covariates were all significant. However, in the Bayesian geostatistical model, the environmental factors did not remain significant. This could be explained by other unmeasured factors such as: access to health care, deforestation (especially the Lake Victoria shores areas), irrigation, swamp draining, etc. Information on some of these factors may be obtained from remote sensing sources. However, information on health related factors would require collection of national and regional data on such variables at high spatial resolution. We observe that in Zanzibar none of the covariates included in the analysis were significantly associated with malaria risk. This is not surprising considering the high level of malaria control implemented on the islands, which counteracted the environmental and climatic effects.

Additional nationally representative surveys were carried out in Tanzania during 2008, namely the NMCP malaria indicator survey (June–July) and the Tanzania National Insecticide Treated Nets Programme (NATNETS) (July–September). Although the three surveys differ in timing and sampling methods, one could compile the data sets and check if the model-based predictions are more accurate, especially in areas where our map shows high uncertainty. If the disease burden estimates would be similar, one could suggest optimizing the resource allocation for conducting a single national survey, instead of three.
